# Mitochondrial oxidative phosphorylation is linked to T-cell exhaustion

**DOI:** 10.18632/aging.103995

**Published:** 2020-09-07

**Authors:** Shuai Jiang

**Affiliations:** 1 Department of Microbiology and Immunology, Louisiana State University Health Sciences Center, Shreveport, LA 71130, USA

**Keywords:** mitochondrial oxidative phosphorylation, T cell exhaustion, NFAT, N-acetylcysteine

Mounting evidence indicates that metabolic reprogramming is not only a hallmark of tumor cells, but also critical for various immune cells, including T cells [1]. Studies of immunometabolism demonstrate that the functional roles of T-cell subsets are linked to different metabolic pathways and key factors (e.g., metabolic enzymes and metabolites), which can modulate T-cell development, differentiation, and activation [2]. In certain pathological settings, such as tumors, T-cell populations called “exhausted T cells” are unable to exert their functional roles. This phenomenon is similar to observations in chronic viral infection; however, the underlining molecular mechanism and its corresponding metabolic alterations remains unclear. It is presently unknown how repetitive antigen challenge leads to T-cell exhaustion, as exhibited by self-renewal ability failure. Thus, there is substantial interest in elucidating how metabolism represents a key link to T-cell exhaustion.

**Figure 1:**
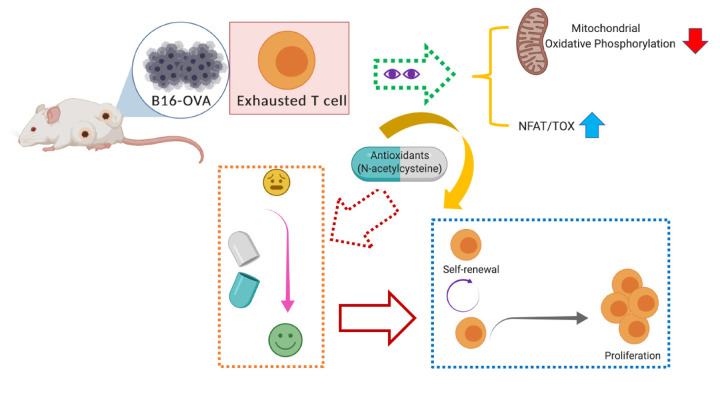
N-acetylcysteine administration reinstates impaired mitochondrial OXPHOS in exhausted T cells, thus restoring the T-cell self-renewal and proliferation capacities in B16F10-OVA-challenged mice.

In the new research article “Impaired mitochondrial oxidative phosphorylation limits the self-renewal of T cells exposed to persistent antigen” [3], Dr. Thompson’s group at Memorial Sloan Kettering Cancer Center characterized the role of metabolic reprogramming in T-cell exhaustion using mouse models of melanoma (Figure 1). They found that persistent antigen challenge led to a shift in metabolic reprogramming, which was critical for restricting T-cell self-renewal and proliferation. The authors observed that exhausted T cells exhibited higher glycolysis, impaired mitochondrial functions, and lower nucleotide biosynthesis. Further characterizing the molecular mechanism of mitochondrial dysfunction in exhausted T cells, they detected significantly enhanced activity of the key regulator of T-cell exhaustion NFAT, and demonstrated that antigen-induced ROS activity was tightly linked to T-cell exhaustion. Moreover, they proved that antioxidant treatment restored metabolic T-cell function and self-renewal in exhausted T cells during chronic antigen stimulation, both *in vitro* and *in vivo*. This study highlights that oxidative force is an aftereffect of chronic antigen-driven mitochondrial dysfunction, which can diminish T-cell proliferation and self-renewal capacities *in vitro* and *in vivo*, suggesting that treatment with a functional sustaining redox balance may restore T cells from “exhausted” to “effective” status.

Previous studies have emphasized the need to further characterize key factors controlling T-cell exhaustion to guide the development of future therapy to enhance anti-tumor immunity. Well-established examples include CTLA-4, PD-1, B7-H4, and CD28 [4]. For example, CD28 signaling can enhance glycolytic flux to satisfy the metabolic needs for T-cell activation [5], whereas PD-1 and CTLA-4 signaling impede T-cell activation by impairing glucose uptake [6]. Overcoming the restriction of T-cell exhaustion would be another critical means of enhancing anti-tumor therapy. In the new study, Vardhana et al. [3] additionally characterized how metabolic alterations influence exhausted T cells, and demonstrated that antioxidants such as N-acetylcysteine could reverse exhausted T-cell capacity and preserve anti-tumor T-cell responsiveness *in vivo*. Unrestrained oxidative stress can result in protein and/or DNA destruction in cancer cells, and antioxidants (e.g., vitamin C and selenium) have been extensively utilized to counter these effects in cancer. However, vitamin C reportedly enhances the expansion of regulatory T cells (Tregs) [7], which decreases anti-tumor immunity. It is possible that N-acetylcysteine could exert side-effects on other immune cell subsets, such as Tregs and dendritic cells (DCs). Thus, it is warranted to further dissect the global alterations of immunity during N-acetylcysteine treatment. Ideally, further research will yield an optimal synergy strategy for boosting anti-tumor immunity against melanoma.

T-cell exhaustion is also reported in patients with hepatitis B virus (HBV) and hepatitis C virus (HCV), and it is unknown whether this occurrence is related to a metabolic mechanism. It would be interesting to analyze exhausted HBV-specific CD8^+^ T cells by liquid chromatography–mass spectrometry (LC–MS) and single-cell RNA sequencing. Non-coding RNAs, such as microRNA-155, reportedly regulate exhausted CD8^+^ T cells in models of chronic lymphocytic choriomeningitis virus (LCMV) infection [8]. It remains unclear how NFAT is modulated in exhausted T cells, and whether microRNA is involved in this process. Several potential microRNAs are predicted to target NFAT1, including microRNA-137 and microRNA-30-5p. Future studies are needed to examine how N-acetylcysteine alters microRNA expressions, and how NFAT and the transcription factor TOX are regulated in exhausted T cells.

The new work by Vardhana et al. [3] connects mitochondrial dysfunction to exhausted T cells using *in vitro* chronic antigen stimulation and *in vivo* mouse models. Their findings indicate a potential new strategy for enhancing anti-tumor immunity against melanoma. Since PD-1/PD-L1 targeting has been utilized in triple-negative breast cancer (TNBC), the murine TNBC cell line 4T1 might be useful for testing the link between mitochondrial oxidative phosphorylation and self-renewal of exhausted T cells upon persistent antigen challenge in future research.
